# Description of the data from the Collaborative Study on the Genetics of Alcoholism (COGA) and single-nucleotide polymorphism genotyping for Genetic Analysis Workshop 14

**DOI:** 10.1186/1471-2156-6-S1-S2

**Published:** 2005-12-30

**Authors:** Howard J Edenberg, Laura J Bierut, Paul Boyce, Manqiu Cao, Simon Cawley, Richard Chiles, Kimberly F Doheny, Mark Hansen, Tony Hinrichs, Kevin Jones, Mark Kelleher, Giulia C Kennedy, Guoying Liu, Gregory Marcus, Celeste McBride, Sarah Shaw Murray, Arnold Oliphant, James Pettengill, Bernice Porjesz, Elizabeth W Pugh, John P Rice, Todd Rubano, Stu Shannon, Rhoberta Steeke, Jay A Tischfield, Ya Yu Tsai, Chun Zhang, Henri Begleiter

**Affiliations:** 1 Department of Biochemistry and Molecular Biology, Indiana University School of Medicine, 635 Barnhill Drive, Indianapolis, IN, USA; 2 Department of Psychiatry, Washington University, 660 South Euclid, St. Louis, MO 63110-1093, USA; 3 Center for Inherited Disease Research, Institute of Genetic Medicine, Johns Hopkins University School of Medicine, Baltimore, MD, USA; 4 Affymetrix, Inc., 3380 Central Expressway, Santa Clara CA 95051, USA; 5 Illumina, Inc., 9885 Towne Centre Drive, San Diego, CA 92121-1975, USA; 6 Department of Psychiatry, State University of New York, Downstate Medical Center, 450 Clarkson Avenue Box 1203, Brooklyn, NY 11203-2098, USA; 7 Department of Genetics, Rutgers University, 604 Allison Road, Piscataway, NJ 08854-8082, USA

## Abstract

The data provided to the Genetic Analysis Workshop 14 (GAW 14) was the result of a collaboration among several different groups, catalyzed by Elizabeth Pugh from The Center for Inherited Disease Research (CIDR) and the organizers of GAW 14, Jean MacCluer and Laura Almasy. The DNA, phenotypic characterization, and microsatellite genomic survey were provided by the Collaborative Study on the Genetics of Alcoholism (COGA), a nine-site national collaboration funded by the National Institute of Alcohol and Alcoholism (NIAAA) and the National Institute of Drug Abuse (NIDA) with the overarching goal of identifying and characterizing genes that affect the susceptibility to develop alcohol dependence and related phenotypes. CIDR, Affymetrix, and Illumina provided single-nucleotide polymorphism genotyping of a large subset of the COGA subjects. This article briefly describes the dataset that was provided.

## Background

Complex diseases, such as alcohol dependence, are influenced by genetic susceptibility, environmental factors, and by interactions among genes and between genes and environment. The Collaborative Study on the Genetics of Alcoholism (COGA) has utilized a multidisciplinary approach, bringing together expertise in many domains to study this complex and important health problem. COGA has been committed to sharing data with researchers in this field to expedite progress in understanding alcoholism and related phenotypes. COGA has also provided data to Genetic Analysis Workshop 11 (GAW11) [1], and has created an archival database of these families, with both phenotypic data and immortalized cell lines; these data are accessible to investigators for further study through NIAAA .

COGA was designed as a family study, incorporating detailed assessments of the participants in many domains to allow derivation and study of endophenotypes along with diagnostic phenotypes. Genome surveys, using microsatellite markers, have been performed on both an initial dataset of 105 multigenerational pedigrees and a replication dataset with 157 multigenerational pedigrees. The results of genome surveys on these datasets have been published [e.g., [2-6]], along with analyses that combined the two [e.g., [7-12]]. Linkage studies of clinical phenotypes and electrophysiological endophenotypes have led to identification of genes involved in brain function as well as genes involved in alcohol dependence and related disorders. COGA has moved beyond identifying regions of linkage and is now identifying individual genes within those regions, using targeted single-nucleotide polymorphism (SNP) genotyping in which multiple SNPs were analyzed for each regional candidate gene. Genes identified include *GABRA2 *[9], *GABRG3 *[12], and *CHRM2 *[10,11].

To test the relative merits of SNPs and microsatellites for localizing genes that contribute to complex diseases and their risk factors, COGA has collaborated with GAW and Center for Inherited Disease Research (CIDR), who enlisted two companies (Affymetrix and Illumina) to generate genome screens using SNPs. CIDR has provided high throughput genotyping of short tandem repeats (STR) markers since 1997, currently providing 11 million STR genotypes per year. As SNP genotyping methods have become more affordable and more amenable to genotyping large numbers of SNPs and samples, CIDR wished to address both a perceived need in the statistical genetics community for additional research related to the analysis of large amounts of SNP data in pedigrees, and the need for CIDR to test high throughput SNP platforms to make an informed decision regarding SNP genotyping services. To address these needs CIDR, in conjunction with Affymetrix and Illumina, provided SNP genotyping of the COGA dataset for GAW14.

The Affymetrix mapping 10k assay [13-15] is an innovative approach that enables rapid typing of 11,560 SNP markers on an array using a single PCR primer and only 250 ng of genomic DNA. The Affymetrix assay uses allele specific hybridization.

The Illumina SNP detection assay [16,17] utilizes allele-specific extension and ligation chemistries. Total genomic DNA is bound to paramagnetic beads. For each SNP, three oligonucleotides are used to interrogate the locus. Two allele-specific oligonucleotides (ASO) each incorporate one of the two possible nucleotides. The third locus-specific oligonucleotide (LSO) anneals 1 to 20 bases downstream of the SNP. This LSO contains a locus-specific address that binds to a complementary address on beads contained in a Sentrix Array Matrix. Specific extension of the complementary ASO occurs joining to the LSO by ligation. Three universal PCR primers are used to amplify the ligated product and incorporate allele-specific fluorescent dyes. Up to 1,536 loci may be multiplexed in one reaction. The Linkage III Panel contains over 4,600 SNP markers distributed evenly across the human genome.

## Methods

### COGA ascertainment and assessment

Initial ascertainment of alcohol-dependent probands (designated Stage I) was performed by screening consecutive admissions at treatment facilities. Probands were assessed with the Semi-Structured Assessment for the Genetics of Alcoholism (SSAGA), a comprehensive diagnostic instrument developed for this study and now widely used [18,19]. Extensive histories of substance use and abuse were gathered along with diagnostic information for multiple Axis I disorders and antisocial personality disorder. To be recruited into the COGA study, probands had to meet both the diagnostic criteria for alcohol dependence (by DSM-III-R criteria [20] and the criteria for definite alcoholism specified by Feighner et al. [21]); thus, the COGA sample is representative of a severely alcohol-dependent population. All first degree relatives of the probands were invited to participate. Children and adolescents in the families were assessed with complementary age-appropriate instruments (C-SSAGA, child and adolescent versions). A set of control families was ascertained to provide normative measures; they were not screened to eliminate those with psychiatric disorders, and are similar to a general population sample. Written informed consent was obtained from all subjects, and the Institutional Review Boards (IRB) of each collaborative site approved all procedures. A more complete description of the recruitment procedures can be found in Begleiter et al. [1,22]. Over 13,000 individuals have been interviewed to date.

A subset of COGA families with at least three alcohol-dependent first degree relatives (designated Stage II) was identified as suitable for a genetic linkage study [1]. These families were extended by diagnostic assessment of more distant relatives in branches reached through an affected member. The Stage II families participated in a more comprehensive multi-domain assessment with an electrophysiologic evaluation of event-related potentials (ERP), event-related oscillations (EROs) and resting electroencephalogram (EEG), endophenotypes associated with alcohol dependence [23,24] that are more proximal to genes and may provide measures of the liability underlying a predisposition to alcohol dependence and related disorders.

### COGA whole-genome survey

A subset of the Stage II families was selected for an initial and a replication genome survey using microsatellite markers. These pedigrees were pruned to eliminate uninformative individuals and branches from the genotyped sample. The initial sample (Wave 1) included 105 multigenerational pedigrees that include 1,214 members, of whom 983 individuals were genotyped. The replication dataset (Wave 2) included 157 multigenerational pedigrees (1,295 individuals).

Microsatellite genotyping started well before standard genome survey sets of markers were available, and therefore markers were drawn from a variety of sources [2]. Initially data were generated manually using agarose gels; later genotyping switched to automated DNA sequencers (ABI373, ABI377). Allele frequencies were estimated with the USERM13 program [25] and the CRIMAP program [26] were used to estimate marker order and distances. Maps were generated from these data.

### GAW 14 dataset

**Figure 1:**
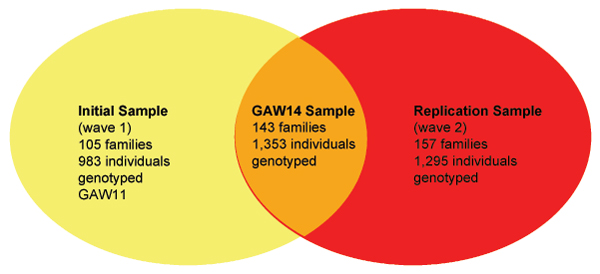
**COGA data subset for GAW 14. **The GAW 14 dataset was drawn partly from Wave 1 and partly from Wave 2.

Limitations on the number of individuals who could be genotyped for GAW14 led us to construct a family sample of 1,353 individuals drawn from both the initial and replication datasets (Figure 1). With non-genotyped individuals included for linking in the pedigrees, the 143 families totalled 1,614 individuals. We selected the sample starting with a core of informative families with at least 6 members who had been interviewed and genotyped, even if they did not have electrophysiological data. Family size ranged from 6 to 30. Other phenotypes forwarded for analysis included alcohol dependence, habitual smoking, and the maximum number of drinks in a 24-hour period. Of the 1,353 individuals selected for genotyping, 1,005 subjects had eyes closed EEG data available, while 905 subjects had Visual Oddball ERP/ERO data available.

It should be pointed out that there are differences in the COGA clinical and electrophysiological datasets to be analyzed by the GAW14 participants and the datasets in the previously published COGA papers [e.g., [4-12]]. These published papers use a greater number of subjects than that provided to the GAW14 participants. Hence, results found by the GAW14 participants will not be identical to those previously published. The reason for the discrepancy in the subject numbers provided to GAW14 is explained in the following points:

1) Due to budgetary constraints, not all of the COGA data were able to be genotyped for GAW14; therefore a subsample of Wave 1 and Wave 2 data was selected for genotyping. This subsample selection was based on large family size and interview status to obtain informative pedigrees. Electrophysiologic measurement was not a criterion for selection. This resulted in the selection of 1,353 subjects for genotyping in GAW14. (Note that the GAW sample consists of 1,614 individuals, because additional non-genotyped individuals were included for linking in the pedigrees).

2) In the COGA project, people who underwent the clinical questionnaire and had blood drawn for genotyping did not always undergo the electrophysiological battery. Only a subset of the total Wave 1 and Wave 2 COGA data have corresponding electrophysiological data available. The subset of the COGA Wave 1 and Wave 2 subjects with resting eyes closed EEG data is 1,553 (as published in Porjesz et al. [8]). The subset of the COGA Wave 1 and Wave 2 subjects with Visual Oddball ERP data is 1337 (as published in Jones et al. [10]).

3) The subset of the COGA dataset selected for GAW14 genotyping and the subset of the COGA dataset with people having electrophysiology data do not overlap completely. This means that out of the 1,353 subjects selected for GAW14 genotyping 1,005 subjects had eyes closed EEG data available, while 905 subjects had Visual Oddball ERP data available.

### CIDR assembly of genotyping plates

For this project, 1,396 samples were received from the COGA DNA and Cell Repository, part of the Rutgers University DNA and Cell Repository. The samples were assigned arbitrary identification numbers. Five percent of the samples were chosen to serve as internal blind duplicates and given new identification numbers. Ninety-two samples and four duplicate DNA samples were placed on each 96-well plate.

DNA was quantified using standard PicoGreen protocols from Molecular Probes. Two of the 96-well plates were randomly chosen for the replication experiment at CIDR. Identical daughter plates were robotically generated (at concentrations appropriate for each technology, 50 ng/μl for Affymetrix and 100 ng/μl for Illumina). Because the performance of the Illumina assay is sensitive to low DNA concentration, replacements for 25 samples that contained less than 50 ng/μl of DNA were requested from Rutgers and included along with the original samples sent to Illumina. Plates were shipped to Affymetrix and Illumina for genotyping in their laboratories.

### Affymetrix SNP genotyping

Affymetrix was supplied with 1,396 samples (the 1,350 COGA samples along with some blind duplicates) to analyze on the GeneChip^® ^Mapping 10k Array [27]. Total genomic DNA was digested with the restriction enzyme *Xba*I, followed by ligation of adaptors. A single primer recognizing the adaptor sequence is used to amplify the ligated DNA fragments and PCR conditions were set to preferentially amplify fragments in the range of 250–1000 bp. The amplified DNA was labeled and hybridized to GeneChip^® ^arrays containing 25-mer DNA probes designed to hybridize with target sequence corresponding to 11,560 SNPs which are known to be located within fragments which will be amplified by the assay.

Each SNP is represented by 40 unique 25-mer DNA probes scattered throughout the array: 20 probes designed against the A allele and 20 against the B allele. Each set of 20 allele-specific probes interrogate the DNA composition at and immediately surrounding the polymorphic site. Relative allele signals are computed from the probe intensities and are used as the input to a classification scheme [28] that produces high-confidence genotype calls for each SNP.

### Illumina SNP genotyping

Illumina received a DNA manifest listing the plate number, well position, and DNA concentration determined using PicoGreen for 1,396 samples. Twenty-five of these samples were below the Illumina concentration specifications. CIDR provided a second submission for each of these low-concentration samples. Both the original and replacement DNAs were genotyped and genotypes were reported for the higher quality sample. Illumina received a revised DNA manifest document listing the plate barcode, well position, concentration, and indicating the replacement relative to the original sample. Illumina received 16 96-well DNA plates containing 1,421 samples that included 25 second aliquots. CIDR placed 92 samples per plate, leaving wells A01–A04 empty for Illumina DNA control samples. Sixteen DNA plates were accessioned into the Laboratory Information Management System (LIMS) using uniquely barcoded plates.

All DNA samples were quantitated in the production lab using PicoGreen. The quantitation results were very similar to those obtained at the CIDR facility. The plates were assigned to the GAW linkage project created in the LIMS database, thus restricting their use to the assays in the Linkage set.

Fan et al. [16] and Gunderson et al. [17] provide a detailed description of the Illumina genotyping platform. All samples were genotyped using the Linkage III Panel containing 4,763 SNP markers. All genotypes were evaluated using a quantitative quality score called GenCall score. A GenCall score ranges from 0 to 1 and reflects the proximity within a cluster plot of the intensities of that genotype to the centroid of the nearest cluster. In addition, we compared the 25 original and replacement paired DNA samples using the GenCall score metric and selected a single sample in each pair of first and second aliquots. Using the GenCall score, we also identified 20 samples with very poor genotyping quality in relation to controls and all other samples. Poorly performing samples were removed from the genotyping report files, and individual genotypes with GenCall scores below 0.25 were assigned a no-call.

### Affymetrix CIDR replicate

Because CIDR hoped to determine if the Affymetrix 10k assay could be used to quickly genotype large numbers of samples, standard Affymetrix 10k protocols were adapted in collaboration with Affymetrix to include automation for all liquid handling steps in a 96-well plate format. Barcode tracking was used to assign plate and well positions to specific Affymetrix GeneChips. Standard Affymetrix protocols were used for chip handling, scanning, and data analysis.

### Illumina CIDR replicate

The replication genotyping experiment performed by CIDR was done using the Illumina BeadLab system. The BeadLab system incorporates automation of all DNA and liquid handling steps. The included LIMS incorporates Illumina's protocols as well as tracking and enforcing workflow. Standard Illumina protocols and reagents were used. Data analysis was performed at CIDR using Illumina's Gentrain and GTS Reports software. The cluster definitions were defined independently on the replicate data set.

### CIDR quality control and data release

Data received from Affymetrix and Illumina were checked for a variety of quality control measures, then combined with COGA family file and formatted for release to GAW14. Quality control calculations included: missing data rates, error rates (based on lack of concordance of the 5% internal blind duplicate and the between lab replicate genotypes), and Mendelian inconsistencies.

## Results

### Affymetrix SNP genotyping

Each sample in the COGA dataset was analyzed with the standard Mapping 10k assay. Of the 1,396 samples supplied, 1,381 yielded enough DNA to analyze [The following 15 samples did not yield enough DNA to genotype: CR1371, CR1315, CR1259, CR1169, CR0959, CR0967, CR0859, CR0789, CR0563, CR0370, CR0269, CR0227, CR0150, CR0047, CR0062]. The median call rate over all 1,381 samples was over 95% with an estimated accuracy of greater than 99%. Two forms of quality control were performed on the samples before the final submission of the dataset to GAW14: calls on the X chromosome were checked against the labeled sex of each sample and families within the study were checked for Mendelian inheritance errors. These quality controls revealed 10 problematic samples (sex or pedigree inconsistencies) [The following 10 samples exhibited gender and/or Mendelian inconsistencies: sex errors: CR1234, CR1112, CR1125, CR1037, CR0728, CR0542; Mendelian inconsistencies: CR1224, CR0221; sex and Mendelian inconsistencies: CR1337, CR0538] in the samples supplied by COGA.

Genetic maps were supplied with the Affymetrix GeneChip^® ^Mapping 10k Array data set to CIDR. SNPs were mapped to unique physical positions on NCBI genome build 34 and interpolated onto one of two framework genetic maps: deCode [29] and Marshfield [30]. Because those framework maps contain multiple microsatellite markers at the same genetic location, interpolation onto these maps can cause non-unique SNP positions. We therefore removed all but one microsatellite marker at each genetic location to create a non-redundant framework, allowing all SNPs with unique physical positions to also have unique interpolated genetic positions [31]. These maps are periodically updated on new versions of the NCBI human genome sequence and are located on the Affymetrix NetAffx website for download [32].

### Illumina SNP genotyping

Production genotyping began on January 16, 2004 and the genotyping report files were delivered on March 5, 2004, a time span of 56 days from the receipt of DNA to data delivery. The files contained the DNA barcode ID, the locus ID, the genotypes, and the GenCall score for each genotype. As genotypes are designated by alleles A and B, an allele key file was provided with context sequence for each SNP and the designation for the nucleotides that represent alleles A and B.

Genotypes were reported for a total of 1,376 DNA samples. Of the 4,763 SNP markers, Illumina reported genotypes for 4,752 resulting in a locus conversion rate of 99.77%. In addition, genetic map positions for each SNP were provided from observed meiotic recombination in 28 CEPH reference pedigrees as described in Murray et al. [33].

### CIDR quality control and data release

Currently the laboratory methods used for the 10k Affymetrix assay include multiple manual steps involving the movement of DNA and reagents via a single-channel or multi-channel pipettes. As a result, one sample swap occurred in the CIDR lab. This sample problem was detected when checking for Mendelian inconsistencies, sex, and replicate errors. Identity by state sharing was calculated within families and across all samples in the dataset as an additional method to screen for problematic samples. Suspect samples were re-genotyped. Confirmation that the problems were resolved was achieved by checking lab-to-lab and within-lab replicates as well as confirming genotype concordance for 26 SNPs in common between the Affymetrix and Illumina datasets.

Two versions of the data were provided to GAW (Tables 1 and 2): raw data and clean data with Mendelian inconsistencies removed. Genotyping data was ordered using the maps provided by the companies and merged with the COGA family data to make the raw comma delimited data files. PEDCHECK [34] was used to detect Mendelian inconsistencies for each SNP. Level 0 and 1 checks were run, and Mendelian inconsistencies were removed in each nuclear family according to the rules listed below:

1) If a parent or two parents are inconsistent with a child, the genotype of the child will be zeroed out.

2) If a specific parent is inconsistent with more than 1 child, the genotype of that specific parent will be zeroed out.

3) If two parents are inconsistent with more than 2 children, the genotypes of the nuclear family will be zeroed out.

After removing Mendelian inconsistencies, files in the format of MERLIN and Linkage PRE-MAKEPED were generated for each chromosome with 250 SNPs per file. SNPs within all chromosome data files were ordered according to the genetic map.

## Conclusion

Because the families and individuals selected for genotyping as part of GAW14 were a subset of families from both the initial and replication datasets used for COGA's published analyses, we do not expect that results will be identical to those previously published. This Genetic Analysis Workshop provides a remarkable opportunity to compare genome surveys using microsatellites to those using SNPs, in a very rich dataset that has both qualitative (e.g., diagnosis) and quantitative (e.g., electrophysiological) phenotypes reflecting a common, complex disease, alcoholism. We hope that the data we have provided will serve as a stimulus for progress in the genetic analysis of complex diseases.

## Abbreviations

ASO: Allele-specific oligonucleotide

CIDR: Center for Inherited Disease Research

COGA: Collaborative Study on the Genetics of Alcoholism

EEG: Electroencephalogram

ERP: Event-related potentional

ERO: Event-related oscillation

GAW: Genetic Analysis Workshop

IRB: Institutional Review Boards

LIMS: Laboratory Information Management System

LSO: Locus-specific oligonucleotide

NIAAA: National Institute of Alcohol and Alcoholism

NIDA: National Institute of Drug Abuse

SNP: Single-nucleotide polymorphism

SSAGA: Semi-Structured Assessment for the Genetics of Alcoholism

STR: Short tandem repeat

## Authors' contributions

COGA authors (HJE, LJB, TH, KJ, BP, JPR, JAT, HB) were responsible for the ascertainment, assessment, initial DNA preparation, and microsatellite genotyping. CIDR authors (PB, KFD, JP, EWP, YYT) were responsible for all DNA sample formatting and distribution, genotyping of replication sample subset on each platform at CIDR, quality control of all SNP data, and formatting of all SNP data released to GAW 14 participants. Affymetrix authors (MC, RC, MK, GCK, GL, GM, SC, CZ) and Illumina authors (MH, CM, AO, SSM, TR, SS, RS) were responsible for the SNP genotyping done with their platforms.

## Figures and Tables

**Table 1:** Summary statistics: GAW14 dataset

	Illumina III	Affymetrix 10k
Total samples	1,376	1,380
No. SNPs	4,752	11,560
Total genotypes	6,538,752	15,952,800
Missing data rate	0.05%	5.25%
Error rate	0.005%	0.052%
Inconsistency rate	0.09%	0.12%

**Table 2:** Summary statistics: CIDR independent replicate data

	Illumina III	Affymetrix 10k
Total samples	192	192
No. SNPs	4,782	11,560
Total genotypes	875,106	2,127,040
Missing data rate	0.13%	6.05%
Between lab consistency rate	99.95%	99.91%*

*Error rate after correction of one laboratory sample swap
